# Recent Progress in Modification of Polyphenylene Oxide for Application in High-Frequency Communication

**DOI:** 10.3390/ma17051086

**Published:** 2024-02-27

**Authors:** Lingyuan Liao, Wenhong Ruan, Mingqiu Zhang, Musong Lin

**Affiliations:** 1 Key Laboratory for Polymeric Composite and Functional Materials of Ministry of Education, GD HPPC Lab, School of Chemistry, Sun Yat-sen University, Guangzhou 510275, China; liaoly5@mail2.sysu.edu.cn (L.L.); ceszmq@mail.sysu.edu.cn (M.Z.); 2 Guangdong Provincial Laboratory of Chemistry and Fine Chemical Engineering Jieyang Center, Jieyang 515200, China; 3 Electric Power Research Institute of Guangdong Power Grid Corporation, Guangzhou 510080, China; linmusong@163.com

**Keywords:** polyphenylene oxide, modification, dielectric properties, circuit substrate, high performance

## Abstract

With the rapid development of highly integrated electronic devices and high-frequency microwave communication technology, the parasitic resistance–capacitance (*RC*) delay and propagation loss severely restrict the development of a high-frequency communication system. Benefiting from its low dielectric constants (*D_k_*) and low dielectric loss factor (*D_f_*), polyphenylene oxide (PPO) has attracted widespread attention for its application in the dielectric layers of integrated circuits. However, PPO suffers from a very high melting viscosity, a larger coefficient of thermal expansion than copper wire and poor solvent resistance. Recently, many efforts have focused on the modification of PPO by various means for communication applications. However, review articles focusing on PPO are unexpectedly limited. In this article, the research progress concerning PPO materials in view of the modification of PPO has been summarized. The following aspects are covered: polymerization and design of special chemical structure, low molecular weight PPO and blending with thermosetting resin, hyperbranched PPO, thermosetting PPO and incorporating with fillers. In addition, the advantages and disadvantages of various types of modification methods and their applications are compared, and the possible future development directions are also proposed. It is believed that this review will arouse the interest of the electronics industry because of the detailed summary of the cutting-edge modification technology for PPO.

## 1. Introduction

In recent years, with the continuous downsizing of electronic devices as well as the rapid development of high-frequency communication technology, the parasitic resistance–capacitance (*RC*) delay and propagation loss have become the bottlenecks that restrict the development of electronic instruments [1,2,3,4]. The calculation formula for the *RC* delay is shown in Formula (1) [5].

(1) $$RC=2\rho D_{k}(\frac{{4l}^{2}}{P^{2}}+\frac{l^{2}}{T^{2}})$$

The *l*, *T* and *ρ* represent the length, thickness and specific resistance of the conductor, respectively. *P* is the distance between two conducting lines. To reduce the size of electronic devices while enhancing their performance, it is necessary to shorten the distance between two conducting lines (*P*). Therefore, this change leads to an increase in the *RC* delay.

Additionally, the signal propagation loss (*L*) is proportional to *f*, *D_f_* and the square root of *D_k_*, as shown in Formula (2) [6].

(2) $$L=k\times (f/c)\times D_{f}\times \sqrt{D_{k}}$$

The *c* represents the light speed, *f* is frequency, *D_k_* is dielectric constant and *D_f_* is the dielectric loss factor of the interlayer dielectric materials, respectively.

It is obvious that the *RC* delay increases with the shrinking size of integrated circuits, and the propagation loss increases with the increase in frequency. To reduce the signal transmission delay and propagation loss, one of the most efficient ways is to use materials with low *D_k_* and low *D_f_* in communication devices, especially in military, aerospace, artificial intelligence, autopilot, and internet of things [7,8].

A printed circuit board (PCB), as part of an integrated circuit, is composed of a polymeric matrix reinforced with glass fabrics and copper metal circuit. It has a significant impact on signal transmission [9,10,11]. A PCB should have the characteristics of low *D_k_* and low *D_f_* to ensure high-speed, lossless signal transmission within the PCB [12]. Moreover, the PCB serves the function of supporting and connecting circuit components. Therefore, they also need to meet certain requirements for circuit component installation and operation. On the one hand, more densely drilled holes and solder are required in the high-density multilayer installation of electronic components on PCBs, which puts forward a requirement that the polymeric matrix of PCBs must endure high-temperature lead-free reflow soldering and chemical solvent washing to remove soldering residues [13]. On the other hand, the ever-increasing power density of these devices generates heat during operation, resulting in a mismatch of the coefficient of thermal expansion (CTE) values between the polymeric matrix and copper conductor, which may cause thermal stress and result in various defects [14]. To avoid that situation, the polymeric matrix should possess low CTE values close to the copper conductor (18 ppm/°C) and good heat dissipation performance [15,16]. Overall, the polymeric matrix used in a high-frequency and high-speed PCB should not only have the advantages of traditional materials, such as mechanical strength, resistance to chemical corrosion, and electric insulation, but also satisfy some new demands, including excellent dielectric performance, dimensional stability and heat dissipation performance. The conventional resins, such as epoxy resin and phenolic, cannot satisfy the demands of the current development of low *D_k_* and low *D_f_* in high-frequency and high-speed applications. Therefore, developing a material that meets the requirements of high-frequency and high-speed applications becomes very important.

Polyphenylene oxide (PPO) possesses low *D_k_* (2.5), low *D_f_* (0.002~0.003), and low moisture absorption [17], and it has garnered more attention from academics and industry in recent years. However, there are several drawbacks that hinder its application. First, as a thermoplastic resin, PPO is unable to meet the requirements of the PCB manufacturing processes due to its low service temperature and poor solvent resistance. Second, thermoplastic PPO has a much higher CTE value (76 ppm/°C) than that of conductive copper [18]. Moreover, PPO has a high melt viscosity, poor mobility, and poor processability, which seriously impact the application of PPO [19]. Over the past few decades, many researchers have focused on preparing PPO with outstanding comprehensive properties via various modification strategies. These strategies include grafting PPO with reactive groups to improve dimensional stability, designing PPO with a hyperbranched structure to reduce melt viscosity, redistributing high molecular weight PPO into low molecular weight to improve the compatibility between PPO and other resins, and preparing PPO through special monomers or ameliorative methods. However, a comprehensive overview of this area is rare. In this article, we review recent studies on the modification of PPO for high-frequency communication devices. We begin by introducing the polymerization mechanism and special structures of PPO. Then, we move to low-molecular-weight PPO and its incorporation with thermosetting resin. Next, we delve into hyperbranched PPO and thermosetting PPO. Subsequently, we focus on PPO-based composites modified by inorganic fillers. Finally, we discuss the promise, issues and future directions of PPO as a polymeric matrix for high-frequency and high-speed PCBs.

## 2. Polymerization of PPO and Its Chemical Structure

**Figure 1:**
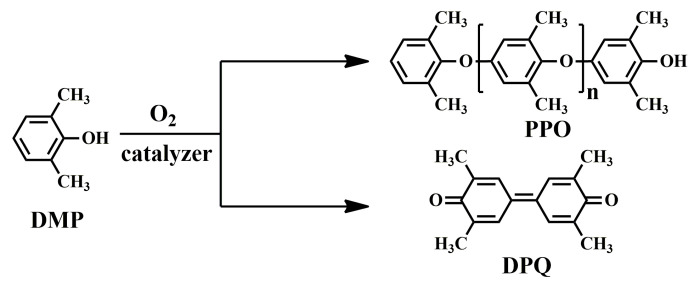
Reaction scheme for the oxidative coupling of DMP to PPO and DPQ.

In 1956, Hay found that when 2,6-dimethylphenol (DMP) was dissolved in pyridine solution with oxygen and cuprous chloride at room temperature, an oxidative coupling reaction occurred, resulting in the solution rapidly becoming very viscous [20]. After separation, a linear polymer with high molecular weight, later called polyphenylene oxide (PPO) or polyphenylene ether (PPE), was obtained, along with small amounts of the C–C coupled diphenoquinone (DPQ) product. The synthetic route of PPO with regular chemical structure is shown in Figure 1 [21].

**Figure 2:**
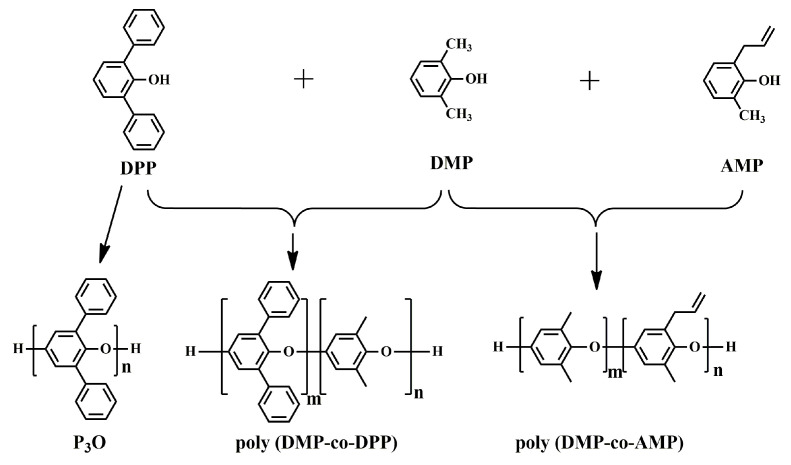
Oxidative polymerization reaction scheme of P_3_O, poly(DMP-co-DPP) and poly(DMP-co-AMP).

In addition to DMP, the oxidative coupling reaction was also applied to other 2,6-disubstituted phenols, such as 2,6-diphenylphenol (DPP) and 2-allyl-6-methylphenol (AMP), to obtain PPO with a nonregular chemical structure, as shown in Figure 2. Hay et al. synthesized completely aromatic poly(phenyleneoxide)s (P_3_O) using DPP as raw material and bidentate amines as the ligand for the copper salt through the oxidative coupling reaction [22,23]. Different from PPO, which hardly crystallizes and behaves as an amorphous polymer, P_3_O is easy to crystallize, resulting in a high glass transition temperature (*T_g_*) of 230 °C and melting point (*T_m_*) of 480 °C (measured by DSC). Moreover, P_3_O has a low *D_k_* of 2.76, a low *D_f_* of 2.5 × 10^−4^ at 50 Hz, and excellent hydrolytic stability. However, due to its crystallization behavior and high *T_m_*, P_3_O requires high melt processing temperatures, which makes it susceptible to thermal degradation reactions. For these reasons, the commercialization of P_3_O was never carried out beyond the pilot plant stage [24]. Yang et al. developed partially aromatic crosslinked poly(2,6-dimethylphenol (95 mol%)-co-2,6-diphenylphenol (5 mol %)) from DMP and DPP through an oxidative coupling reaction, followed by curing with crosslinking agents. The crosslinked poly(DMP_95_-co-DPP_5_) exhibited a high *T_g_* of 250 °C (measured by DMA), a low *D_k_* of 2.6 and a low *D_f_* of 4 × 10^−3^ at 10 GHz. Fukuhara prepared a thermosetting poly(2-allyl-6-methylphenol-co-2,6-dimethylphenol)s (poly(DMP-co-AMP)) through oxidative coupling copolymerization of AMP with DMP and followed by thermal curing at 300 °C [25]. The cured copolymers exhibited excellent solvent resistance, high *T_g_* (229~235 °C), low *D_k_* (2.5~2.6), and the low *D_f_* (1.5~1.9 × 10^−3^) at 1 MHz. Afterwards, Jun Nunoshige et al. blended poly(DMP-co-AMP) with 1,2-bis(vinylphenyl)ethane (BVPE). BAPE accelerated the cross-linking reaction of the allyl group in poly(DMP-co-AMP) and therefore reduced the curing temperature. Moreover, the resulting polymer blend showed lower *D_k_* (2.39~2.40) and *D_f_* (0.0013~0.0019) as the BVPE content increased [26].

The 2,6-disubstituted phenols with different substituents indeed exhibit a wide range of material properties. However, as the substituents become larger and bulkier, the resulting molecular weight and yield of linear polymers decrease. Consequently, the C–C coupled DPQ becomes the predominant product. For example, when the substituents are t-butyl groups, only the DPQ compound is formed.

## 3. Low-Molecular-Weight PPO

PPO possesses several desirable properties, including low *D_k_*, low *D_f_*, and low moisture uptake. These characteristics make it an excellent additive for reducing the *D_k_* and *D_f_* of polymers commonly used in electronic substrates, such as epoxy resin and cyanate resin. However, high-molecular-weight PPO resin has some drawbacks, including low reactivity and poor miscibility with other resins, such as epoxy and cyanate resin, primarily because of the lack of adequate phenolic end groups. There are some reasons for the lack of phenolic end groups. First, the phenolic end group functionality of high-molecular-weight PPO should be equal to one in theory. However, in practice, it is lower than one due to the presence of Mannich base-type end groups generated during the oxidative polymerization of DMP. These Mannich base groups exhibit lower reactivity compared to normal phenolic end groups. In addition, the higher the molecular weight, the lower the phenolic end group content. Based on the above analysis, reducing the molecular weight of PPO and increasing the phenolic end group functionality would be beneficial for improving the reactivity and compatibility between the PPO and these resins.

**Figure 3:**
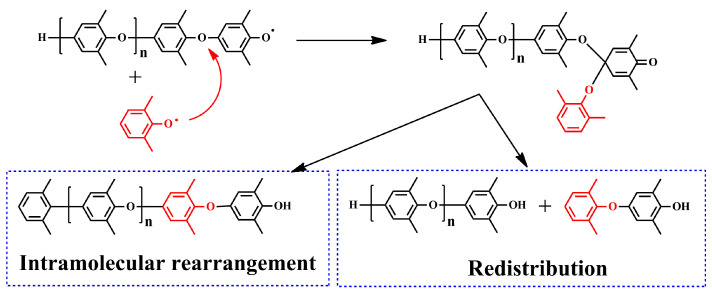
The reaction mechanism for the polymerization of PPO.

Redistribution of PPO is a preferable method to obtain low-molecular-weight PPO with the desired end groups. Redistribution of PPO was discovered during the study of the polymerization mechanism of PPO, which is a side reaction in the synthesis of PPO. Hay et al. found that the polymerization degree was very low in the initial stage of the polymerization reaction of PPO, but the polymerization degree suddenly increased in the later stage, which is significantly different from chain polymerization initiated by free radicals [20]. Except for the chain-extending reaction, they speculated that there exists an equilibrium reaction that maintains PPO oligomers at a low polymerization degree between PPO oligomers and monomers, and then PPO oligomers convert to high-molecular-weight PPO suddenly in the later stage. Bolon et al. have confirmed this speculation through experiments, showing that the PPO oligomers can polymerize to high-molecular-weight PPO in the presence of initiators [27]. The reaction mechanism is shown in Figure 3.

**Figure 4:**
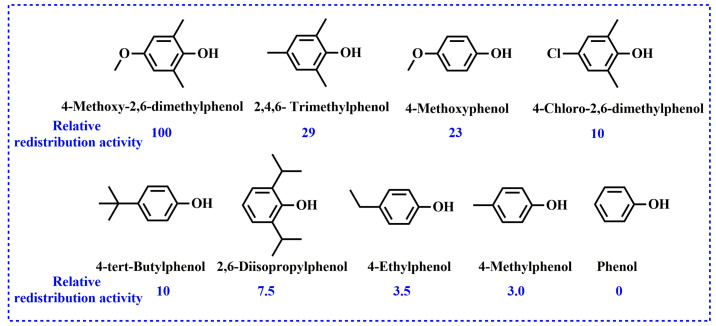
Redistribution activity of phenols compared to 4-methoxy-2,6-dimethylphenol.

According to above realization, upon the addition of phenolic compounds to a high-molecular-weight PPO, the redistribution reaction can occur and yield low-molecular-weight PPO (r-PPO). The mechanism of the redistribution reaction of PPO is well-studied. Actually, the type of phenolic compound has an effect on the redistribution reaction. White et al. compared the redistribution activity of various phenolic compounds based on the molecular weight of r-PPO and found that phenols containing strong electron withdrawing or high steric hindrance groups, such as p-nitrophenol, p-hydroxyphenyl acetonitrile and 2,6-diphenylphenol, had low redistribution activity [28]. Afterwards, Bolon et al. systemically summarized the relative redistribution activity of phenols (as shown in Figure 4) and drew the conclusion that phenols containing electron-donating substituents have higher redistribution activity [27].

**Figure 5:**
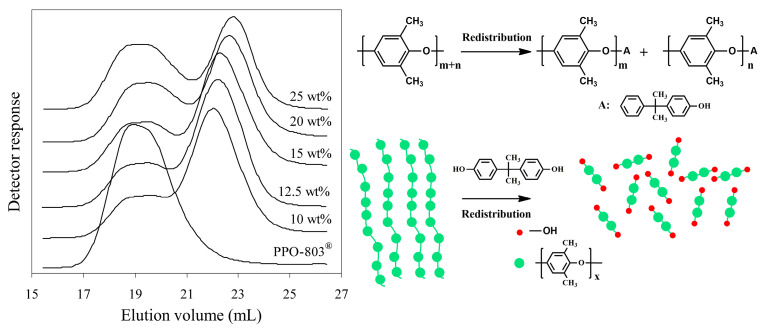
GPC molecular weight distribution of the PPE-2OH products made with different amounts of TMBPA [29].

The content of phenolic groups increases as the molecular weight decreases, but the phenolic end groups’ functionality cannot increase unless the phenols with a bisphenol hydroxyl group are selected to react with PPO. Krijgsman [29] prepared bifunctional PPO-2OH by the redistribution of high-molecular-weight commercial PPO (*M_n_* = 11,000 g/mol) with tetramethyl bisphenol A (TMPBA) in the presence of tetramethyl DPQ. The GPC results showed that the product has a bimodal molecular weight distribution, and only 70–80% of high-molecular-weight PPO is depolymerized to low molecular weight (2000 g/mol), as shown in Figure 5. This is because partial PPO has Mannich base-type end groups, which exhibit lower reactivity and fail to react with phenolic compounds. Similar phenomena also have been reported in other studies [29,30,31,32].

**Figure 6:**
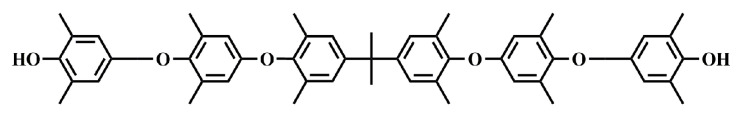
Chemical structure of SA90.

Apart from the redistribution method, the low-molecular-weight PPO can also be prepared through the bottom-up method. Wilhelm et al. proposed a method that simultaneously involves oxidative coupling of DMP and tetramethyl bisphenol A to prepare low-molecular-weight PPO with double terminated hydroxy in the presence of a copper–ammonium catalyst [33]. NORYL SA90 (*M_n_* = 1600 g/mol, the structure is shown in Figure 6), a commercially available PPO, was prepared through this method.

Due to a decrease in the molecular weight, the *T_g_* of r-PPO was decreased. According to the Fox and Flory Formula (3) [34]:

(3) $$T_{g}=T_{g\infty }-\frac{C}{M_{n}}$$

where *T_g_* is the glass transition temperature of a polymer of infinite chain length (equal to 490 *K*), *M_n_* is the number-average molar mass, and *C* is a constant related to the volume of the chain ends (equal to 12.73 × 10^4^ g K/mol) [35].

**Figure 7:**
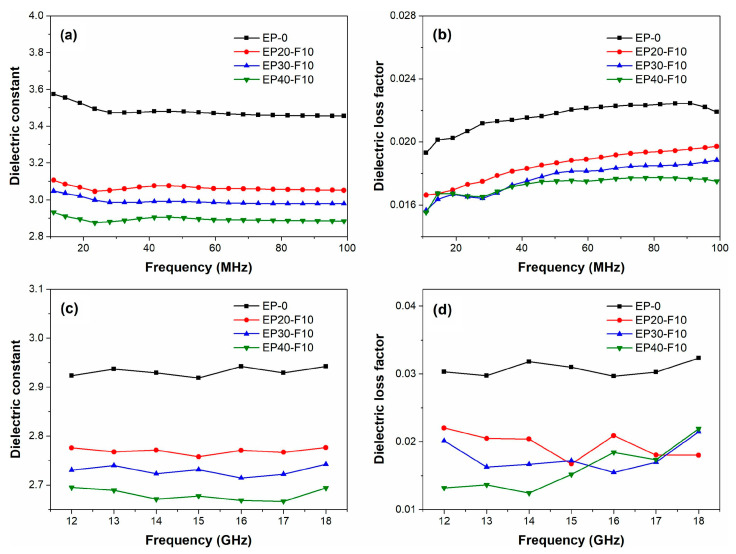
Frequency dependence of the dielectric properties of EP and EP/F-rPPO: (**a**) dielectric constant and (**b**) dielectric loss factor at 10~100 MHz; (**c**) dielectric constant and (**d**) dielectric loss factor at 12~18 GHz [32].

The molecular weight of commercialized PPO is about 20,000~30,000 g/mol, with *T_g_* ranging from 208 to 212 °C. After the redistribution reaction, the molecular weight of PPO is reduced to 2000~5000 g/mol, with *T_g_* ranging from 152 to 190 °C. Moreover, PPO with a low molecular weight is quite brittle. Therefore, low-molecular-weight PPO is rarely used alone but blended with other thermosetting resins. Ling et al. synthesized r-PPO via the reaction of commercial PPO with BPA [36]. Subsequently, this r-PPO was blended with cyanate resin (CE). The results showed that the redistribution reaction not only affects the molecular weight distribution of r-PPO, but also increases the phenolic groups, which can react with cyanate resin to produce cured r-PPO/CE mixed resin systems. The cured r-PPO/CE mixed resin systems exhibited a low *D_k_* of 3.85 and a low *D_f_* of 2.64 × 10^−3^ at 10 MHz. To reduce the *D_k_*, Zhou et al. synthesized a fluorinated redistributed PPO (F-rPPO) with a number-average molecular weight of 3.0~6.2 × 10^3^ g/mol via the redistribution reaction of commercial PPO with 4,4′-(hexafluoroisopropylidene) diphenol (BPAF) using benzoyl peroxide (BPO) as an initiator. F-rPPO was further used to modify epoxy resin [32]. The results showed that the cured EP/F-rPPO resins exhibited improved thermal stability and lower moisture absorption compared to the pristine epoxy resin. Moreover, the *D_k_* and *D_f_* of the cured EP/F-rPPO resins decreased with an increase in the F-rPPO content (see Figure 7) due to the low polarizability of F-rPPO and the large free volume introduced by -CF_3_ groups.

**Figure 8:**
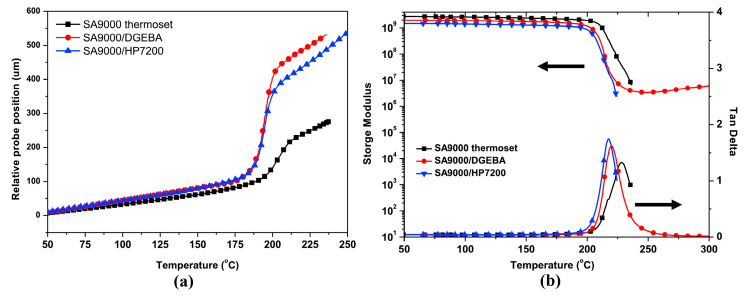
(**a**) TMA curves of SA9000 thermoset and SA9000/epoxy thermosets, and (**b**) DMA curves of SA9000 thermoset and SA9000/epoxy thermosets [37].

The phenolic groups of r-PPO can be further modified by reacting with acyl chloride, anhydride, phenyl ester, and halogenated hydrocarbon to extend its application range. For example, SABIC company (Riyadh, Saudi Arabia) prepared a methacrylate-terminated PPO, named NORYL SA9000, by the esterification reaction of low-molecular-weight PPO (a commercial product, SA 90) with methacrylic anhydride. As shown in Figure 8, SA9000 contains vinyl end groups, which make it more suitable for blending with other unsaturated resins, including styrene, acrylic acid, maleimide, methacrylic acid and epoxy resins. Chen et al. [37] used two commercialized epoxy resins (DGEBA and HP7200) to copolymerize with SA9000. The SA9000/epoxy thermosets show flexibility, high glass transition temperatures of 218~220 °C, low *D_k_* of 2.8~2.9, and extremely low *D_f_* of 3.1~3.2 × 10^−3^ at 1 GHz. However, the CTE increased from 56 ppm/°C (cured SA9000) to 66~67 ppm/°C (SA9000/epoxy), and the *T_g_* values of the SA9000/epoxy are slightly lower than that of cured SA9000, as shown in Figure 8.

**Figure 9:**
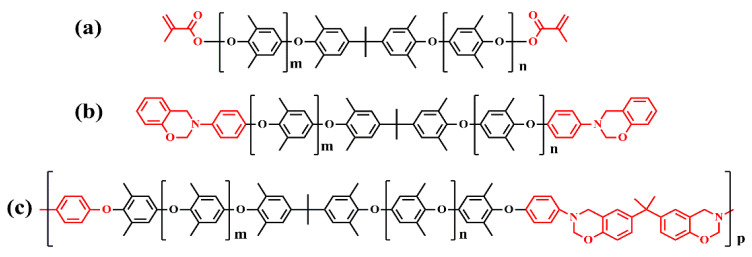
(**a**) Chemical structure of SA9000, (**b**) chemical structure of P-APPO, and (**c**) chemical structure of BPA-APPO [38].

In addition, they prepared an amine end-capped PPO (APPO) via a nucleophilic substitution between phenol-end capped oligo PPO (SA90) and fluoronitrobenzene, followed by catalytic hydrogenation [38]. The amine of the APPO was converted into benzoxazinewere to prepare the PPO containing benzoxazinewere, including telechelic oligomer-type benzoxazine (P-APPO) and the main-chain-type benzoxazine polymer (BPA-APPO). These chemical structures are shown in Figure 9. After that, P-APPO and BPA-APPO were incorporated into dicyclopentadiene-phenol epoxy, respectively, to give the thermoset E-P-APPO and E-BPA-APPO. The thermoset E-P-APPO and E-BPA-APPO show higher *T_g_* (227 and 232 °C) and lower *D_f_* (0.0053 and 0.0050 at 1 GHz) than the SA90/EP (207 °C and 0.006).

Different from high-molecular-weight PPO, low-molecular-weight PPO exhibits low *T_g_* and poor mechanical strength, while it possesses a lot of end phenolic groups. These phenolic groups can be further converted into various reactive groups. Consequently, low-molecular-weight PPO displays good compatibility with various resins. Especially in the field of circuit substrate, it is often copolymerized with thermosetting resins such as EP and CE to decrease the *D_k_* and *D_f_* of these resins.

## 4. Hyperbranched PPO

Hyperbranched polymers, as a class of three-dimensional semispherical dendritic polymer, possess considerably lower viscosity and offer great possibilities for chemical modification due to the abundance terminal functional groups.

**Figure 10:**
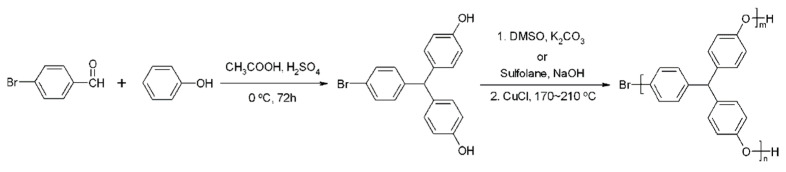
Synthesis of hyperbranched PPO from phenol and p-bromobenzaldehyde [39].

Zhang et al. [39] prepared hyperbranched PPO (HPPO) with terminal phenolic groups by a one-pot polymerization of an AB_2_ monomer, 4-bromo-4′,4”-dihydroxytriphenylmethane, using the modified Ullmann reaction in the presence of DMSO/K_2_CO_3_ or sulfolane/NaOH (as shown in Figure 10). They found that the *T_g_* of the obtained HPPO has no direct connection with the molecular weight but mainly depends on the degree of branching. A high degree of branching (DB) in the molecular architecture reduces the mobility of the chain segments. Moreover, the large number of phenolic terminal groups enhances the polarity and the intermolecular interactions, resulting in increased *T_g_* values for HPPO. These hyperbranched PPOs contain a large amount of phenolic end groups, making them amenable to facile grafting with various functional chain ends, such as methoxy, 1-butoxy, and diethyleneoxy units.

**Figure 11:**
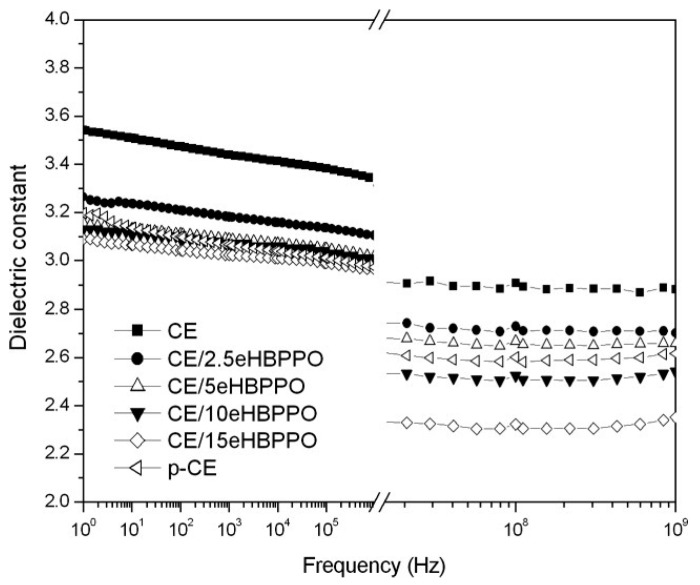
Dependence of the dielectric constant on the frequency for cured CE, p-CE, and CE/eHBPPO resins [40].

Huang et al. [40] synthesized epoxy-functionalized hyperbranched poly(phenylene oxide) (coded as eHBPPO) by converting the peripheral hydroxyl groups of the hyperbranched PPO into epoxy groups. The eHBPPO was then used to modify cyanate resin (CE). The results showed that the cured CE/eHBPPO resins display a lower *D_k_* and *D_f_* than CE resin. Moreover, the dielectric properties of CE/eHBPPO resins were stable in a wide frequency range (1–10^9^ Hz), as shown in Figure 11.

**Figure 12:**
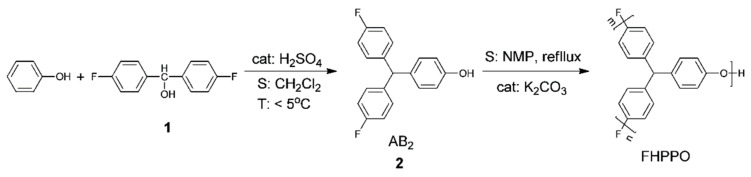
Synthesis route of fluoro-terminated hyperbranched poly(phenylene oxide) [41].

**Figure 13:**
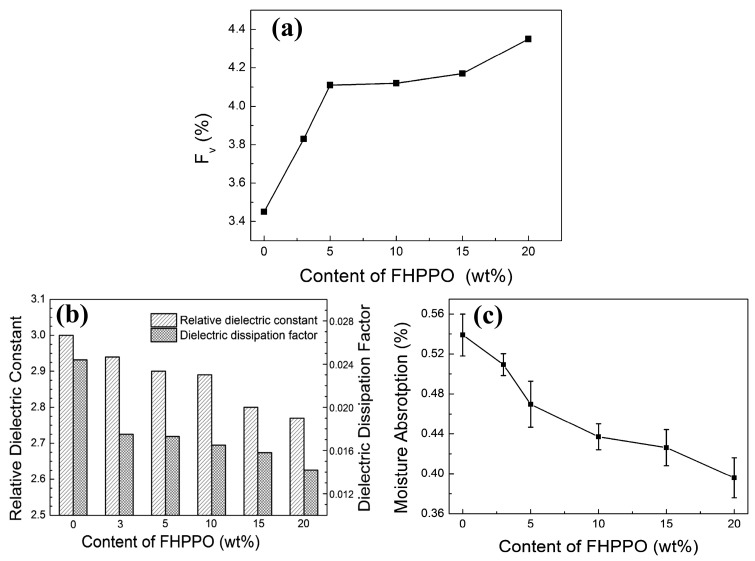
(**a**) The variation of the free volume fractions (Fv) [42], (**b**) moisture absorption and (**c**) dielectric properties of DGEBA/FHPPO composites as a function of the FHPPO loading [41].

Considering the low polarizability and large molar volume of C–F bonds, fluorinated polymers may help to reduce the *D_k_* and *D_f_*. Li et al. [41] prepared fluoro-terminated hyperbranched PPO (FHPPO) from a new AB_2_ monomer, 4-hydroxyl-4′,4”-difluorotriphenylmethane, as shown in Figure 12. The molecular weight (*M_w_*), DB and *T_g_* values of these hyperbranched polymers prepared under different conditions are listed in Table 1. The *T_g_* of the FHPPO increased with the molecular weight and tended to level off at high molecular weights. This can be explained by the coupled effects of two factors. On the one hand, the proportion of rigid triphenyl groups increased with increasing molecular weight, resulting in a limitation of the segments’ mobility. On the other hand, the DB decreased with increasing molecular weight, indicating that more linear structures exist in FHPPOs, leading to a decrease in the *T_g_*. FHPPO was used as a modifier and added to diglycidyl ether of bisphenol A in different ratios to form cured hybrid DGEBA/FHPPO resins. They found that the addition of FHPPO could increase the free volume, reduce moisture absorption, and decrease the *D_k_* and *D_f_* of the cured hybrid materials, as shown in Figure 13a–c.

Presently, studies of HPPO are mainly concentrated on using HPPO to modify other thermosetting resins, such as cyanate resin, epoxy resin and bismaleimide resin, to improve the thermal stability and dielectric properties of these resins. However, there is no report about the dielectric properties of neat HPPO and its relationship with the DB, molecular weight and the type of terminal group. In addition, the method for synthetizing HPPO has suffered the disadvantages of high temperature and long time-consuming nature, which limits its large-scale industrial applications.

## 5. Thermosetting PPO

**Figure 14:**
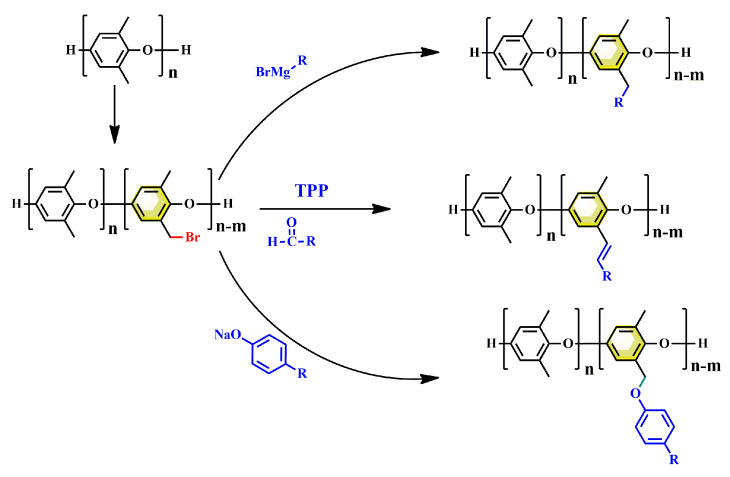
Synthetic route for thermosetting PPO.

Thermosetting resins are more commonly utilized as the polymer matrix of PCBs due to their excellent dimensional stability, solvent resistance, and thermostability compared with thermoplastic resins. However, PPO is a linear amorphous polymer with a highly symmetrical main chain composed of rigid phenolic aromatic rings and methyl groups, without crosslinkable groups. Therefore, many efforts have been made to introduce PPO with reactive groups based on methyl groups. As shown in Figure 14, the modification of PPO via methyl groups mainly involves the following process: (1) brominating of methyl groups; and (2) introducing of a reactive group via various chemical reactions, including nucleophilic substitution, Grignard reaction and so on.

**Figure 15:**
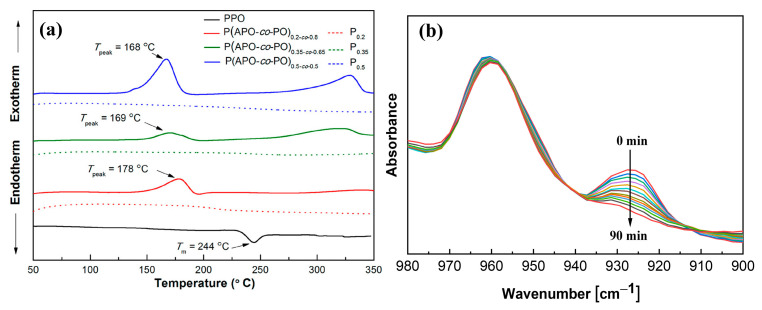
(**a**) DSC traces of PPO and P(APO-co-PO) at a heating rate of 5 °C min^−1^ [45]. (**b**) In situ FTIR spectra of PPO-Allyl-F cured at 250 °C for different curing times [46].

Huang et al. [43] prepared a new epoxidized PPO through the brominating of PPO in halogenated aromatic hydrocarbons, followed by a Wittig reaction, yielding a vinyl-substituted PPO. The vinyl-substituted PPO was then treated with m-chloroperbenzoic acid to form epoxidized PPO with variable pendant ratios. The results showed that both the *T_g_* and the thermal stability were improved with the increase in the epoxide molar content, and the *T_g_* exceeded 300 °C (measured by DSC) when the degree of functionalization was above 30%. Wang et al. [44] have introduced allyl groups into PPO to prepare thermosetting modified PPO (Allyl-PPO) via the reaction between brominated PPO and a Grignard reagent. The cured Allyl-PPO exhibits excellent solvent resistance and a high *T_g_* of 217 °C (determined by DMA loss peak), the *D_k_* of cured Allyl-PPO was 2.84 at 1 MHz. However, the Grignard reagent is inconvenient to operate because of its high sensitivity to moisture. Fang et al. [45] synthesized thermosetting PPO (P(APO-co-PO)) by treating brominated PPO with anethole rather than Grignard reagent, followed by hot-pressing at a high temperature in the presence of a peroxide. The DSC curves indicated that these thermosetting PPO formed crosslinked structures when the temperature was around 168~178 °C (Figure 15a). As a matter of fact, they carried out the curing process at 300 °C. The crosslinked PPO displayed a low *D_k_* of less than 2.74 at 30 MHz, high *T_g_* (more than 220 °C, determined by DSC) and excellent dimensional stability. It is noteworthy that although thermosetting modification of PPO has improved the *T_g_* and dimensional stability, the dielectric properties of thermosetting PPO are worse than those of thermoplastic PPO. This is because grafting of crosslinkable groups in methyl groups inevitably disrupts the symmetrical nature of PPO and increases the dipole moment. Considering the contradiction between improving the dielectric properties and thermosetting of polymer materials, our research group proposed a method to balance thermosetting and dielectric properties by introducing a crosslinking agent with both trifluoromethyl groups and allyl groups to PPO. In situ Fourier transform infrared spectra indicated the resulting PPO with different contents of trifluoromethyl groups was cured at 250 °C to obtain the cured PPO-allyl-F (Figure 15b) [46]. These cured PPO-allyl-F materials exhibited a *D_k_* value in the range of 2.56–2.67 at 10 GHz and a *D_k_* value in the range of 2.57~2.68 at 1 MHz, which were lower than the above-mentioned thermosetting PPO. The density functional theory calculation indicated that though trifluoromethyl slightly increases the polarizability, it provides a large free volume for cured PPO-allyl-F. Therefore, the polarizability per unit volume was decreased, which is beneficial for reducing *D_k_*.

The CTE is also important for the polymer matrix of PCBs. The CTE values of polymers can vary significantly based on their chemical structure, molecular arrangement, and physical state. For instance, highly extended and oriented polymers exhibit low or even negative thermal expansion along the chain axes [47]. Amorphous polymers lack long-range order in their molecular arrangement, so they expand as the temperature rises due to increased molecular vibrations. Moreover, the CTE in thin polymer films is different from that of their bulk counterparts, the anisotropy in CTE stems from the in-plane orientation of polymer main chains generated during film-forming process [48]. PPO is an amorphous polymer, and its polymer chains are not oriented. Additionally, it is not used in the form of thin films. Therefore, researchers seldom consider anisotropy in the CTE of PPO. In the previous literature reports, P(APO-co-PO) and PPO-allyl-F exhibit a lower CTE as the crosslinking density increases [45,46]. This indicates that covalent connections between polymer chains are beneficial for restricting segment movement and free volume expansion.

Thermosetting modification of PPO based on the methyl group on the main chain has the advantage of grafting a large amount of reactive functional groups without reducing the molecular weight of PPO. The thermosetting PPO displays dimensional stability and solvent resistance. However, grafting of crosslinked groups inevitably breaks the symmetrical structure of the PPO, thus increasing the dipole moment, which leads to an increase in the *D_k_*. Although introducing a low polarity and bulk crosslinking group is beneficial for alleviating the adverse effects, it is not resolved completely. How to develop PPO with high-performance, including excellent dielectric properties, dimensional stability, and solvent resistance, remains an open issue.

## 6. PPO-Based Composites

As an effective modification method, incorporating inorganic fillers with certain functionality into the polymer matrix plays a significant role in enhancing polymer performance. Diverse fillers, including carbon material, ceramic material, and metal/metal oxide, have been added into polymers for various purposes, such as electrically conductive, thermal conductive, dielectric properties, electromagnetic interference shielding, and mechanical properties [49,50].

**Figure 16:**
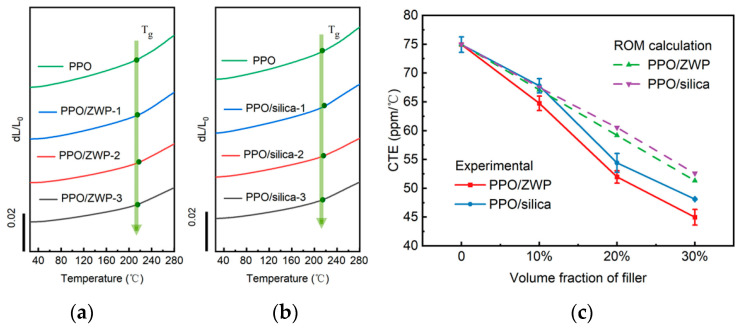
(**a**) TMA curves of PPO/ZWP composites with different ZWP loading (10~30%), (**b**) TMA curves of PPO/silica composites with different silica loading (10~30%), and (**c**) the theoretical and experimental CTE values for PPO, PPO/ZWP composites and PPO/silica composites [52].

To improve the reliability of electronic equipment, it is crucial that the CTE of the polymer used in electronic equipment closely matches that of the other materials, such as silicon (0.5 ppm/°C) and copper wire (18 ppm/°C) [2,51]. The CTE of the material is decided by its composition. Theoretically, the combination of a material with a negative CTE and a material with a positive CTE is expected to obtain composites with a low CTE. Based on this, Zhu et al. [52] incorporated different volume fractions of negative thermal expansion (−3 to −5 ppm/°C) Zr_2_WP_2_O_12_ (ZWP) particles into PPO. To prevent the agglomeration of inorganic fillers and enhance the interaction between the fillers and matrix, KH-570 (3-(Trimethoxysilyl) propyl methacrylate) was used to modify the ZWP. The resulting PPO/ZWP composites, obtained by hot press, exhibited a decreased CTE with increasing filler content. Moreover, these PPO/ZWP composites exhibited a lower CTE than that of PPO/silica composites with the same filler content, as shown in Figure 16.

**Figure 17:**
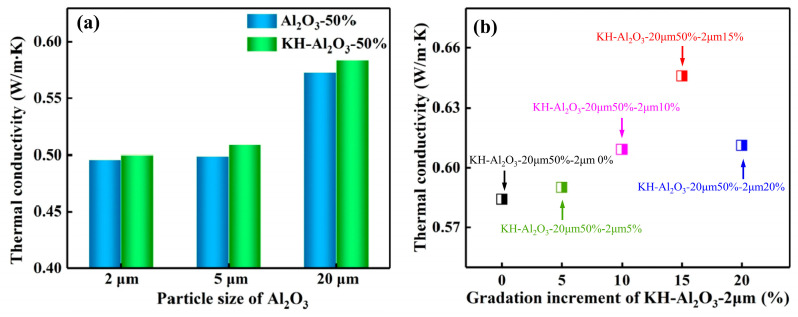
The thermal conductivity of the composite: (**a**) filled with a different particle size of Al_2_O_3_ before and after KH-560 modification at 50 vol%, and (**b**) filled with KH-Al_2_O_3_-20 μm at 50 vol% and KH-Al_2_O_3_-20 μm at x vol% (x = 0–20) [53].

Adding high thermal conductivity fillers into polymers is a common approach to elevate the heat dissipation capability and service life of electronic equipment. Zhang et al. [53] blended gradation-mixed Al_2_O_3_, which was modified by silane coupling agent, into PPO to prepare an insulative layer in copper-clad laminates. They found that the Al_2_O_3_ fillers modified with the silane coupling agent displayed better dispersibility in composites and a lower water absorption rate than that of composites loaded with unmodified Al_2_O_3_ (see Figure 17a). In addition, the gradation-mixed Al_2_O_3_ displayed a better improvement in thermal conductivity (see Figure 17b). This improvement is attributed to the small-size Al_2_O_3_ particles that filled the interval space between the larger ones, thus establishing a more complete heat conduction network.

**Figure 18:**
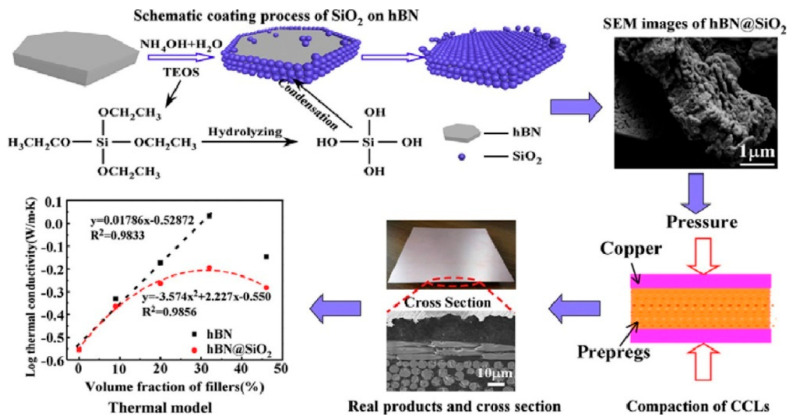
Schematic coating process of SiO_2_ nanoparticles on h-BN and preparation process of CCLs [59].

Hexagonal boron nitride (h-BN) also received attention due to its unique properties, including its mechanical strength, high thermal conductivity (600 W/(m•K)), low *D_f_* (0.0002), and excellent insulation [54,55,56,57,58]. Ge et al. [59] introduced h-BN into the PPO to prepare an insulative layer of copper-clad laminates used in high-frequency applications. SiO_2_ was coated on the surface of h-BN to improve the fluidity of the lamellar h-BN filler, as shown in Figure 18. The resulting CCLs exhibited a high thermal conductivity up to 1 W/(m•K) and a low dielectric loss of 4 × 10^−3^ at 48 wt% h-BN loading, validating the high potential of this composite for use in high-frequency PCBs. However, the thermal conductivity of amorphous silica was much lower than that of h-BN. The h-BN coated with silica displayed a lesser improvement in thermal conductivity compared to uncoated h-BN.

Incorporating fillers into PPO endows composites with specific functions. Notably, the properties of composites were extremely determined by the types of the fillers, the dispersion or orientation of the fillers, and the interfacial interactions between the fillers and polymer [60,61]. Moreover, excessive filler addition to composite tends to decrease the performance, affecting the strength, toughness and dielectric properties. This effect is particularly pronounced in high-frequency and high-power circuit substrates, where the trade-off between low *D_k_* and high thermal conductivity presents a challenge. The tailoring of polymer/filler interfaces, inducing filler orientation and building thermal conductivity pathways play a key role in achieving high-performance materials with desirable dielectric and thermal properties.

## 7. Conclusions

In summary, this review systematically focuses on the modification of PPO for application in a circuit substrate. This includes the structure design of PPO, copolymerization or blending with thermosetting resins, and PPO-based composites. Low-molecular-weight PPO and hyperbranched PPO contain many phenolic groups, which can be converted into various reactive groups. These PPO are used as modifiers and added into multiple thermosetting resins to improve the dielectric performance and thermal stability. By grafting reactive groups into the methyl groups of the main chain, the thermoplastic PPO can be converted into thermosetting PPO, thus improving its solvent resistance, dimensional stability, and thermal stability. Incorporating fillers with specific properties, such as negative thermal expansion, high thermal conductivity, or low *D_f_*, further improves the performance of PPO-based composites.

With the rapid development of integrated, high-power microelectronic devices and 5G technology, PPO has attracted more and more attention for its outstanding properties, thermal stability, and moisture absorption. However, there remain a lot of issues that must be resolved to comply with the development trend of future electronic devices.

(1) The contradiction between reducing the *D_k_* and improving the dimensional stability of polymer materials due to their different requirements for the molecular structure. The relationship between the molecule structure, dielectric, and dimensional stability should be further revealed by subtly structural design.
(2) Another contradiction arises between reducing the *D_k_* and improving the high thermal conductivity of PPO base composites. This is because the *D_k_* of thermal-conducting fillers is usually higher than PPO. Therefore, the pivotal problem is how to improve thermal conductivity at low filler loading. Several approaches can help resolve this issue, such as improving intrinsic thermal conductivity of PPO through designing chemical structure or regulating the condensed state of PPO, building the thermal conduction pathway in composites, improving interface interactions, and reducing phonon scattering at the interface.
(3) High-frequency bands, ranging at 5 GHz or even millimeter waves frequencies (>30 GHz), are used in 5G communication technology. However, many studies provide dielectric properties at low-frequency bands (<1 GHz) by the parallel plate capacitance method. Moreover, the parallel plate capacitance method can be challenging to measure accurately, especially for low-loss materials [62]. Therefore, adopting a high-accuracy method to measure the dielectric properties of materials at high-frequency bands (~GHz) is necessary.

## Figures and Tables

**Table 1:** Properties of hyperbranched PPO in the literature.

Samples	Reaction Conditions	*T_g_* (°C)	*M_w_* (Da)	Polydispersity Index	Degree of Branching	References
HPPO	DMSO/K_2_CO_3_ 170 °C for 32 h	153	2230	1.39	0.71	[39]
HPPO	sulfolane/NaOH 200–210 °C for 6 h	130	5530	2.04	0.48	[39]
FHPPO	NMP/ K_2_CO_3_ 202 °C for 2–5 days	135	2000	1.7	0.63	[41]
		147	2500	2.2	0.60	
		156	5400	3.2	0.56	
		163	5800	3.7	0.55	
		164	6800	4.8	0.53	
